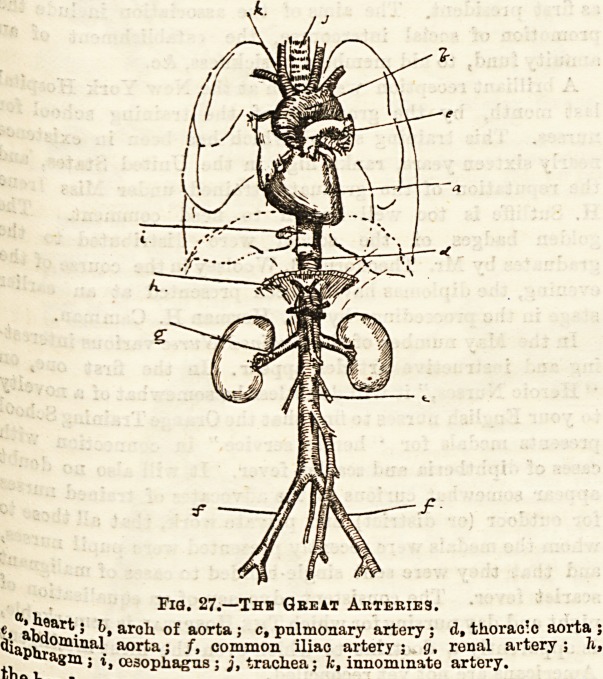# The Hospital Nursing Supplement

**Published:** 1895-05-18

**Authors:** 


"The Hospital" Nursing Mirror.

Being the Extra Nursing Supplement of "The Hospital" Newspaper.

[Contributions for this Supplement should be addressed to the Editor, The Hospital, 428, Strand, London, W.C., and should have the word "Nursing" plainly written in left-hand top corner of the envelope.]

### News from the Nursing World.

### INVALID CHILDREN.

The annual meeting of the Invalid Children's Aid Association was very well attended, the beautiful room lent by Lord Brassey being filled with subscribers and their friends. An urgent appeal was made by the committee both for funds and for workers to carry out the aims of the society. It was reported that many more ladies were needed as regular visitors to the invalid and crippled children on the books of the association, the demand for surgical appliances and other help being also incessant. The funds of the society should materially benefit by the appeals made by Sir William Broadbent, M.D., who presided at the annual meeting, Canon Shuttleworth, and the Bishop of Emmaus, who testified warmly to the work of the association. In the course of last year 795 cases were dealt with, 321 being paid for in homes, and surgical appliances provided for 171. Without increased help it will be impossible for the association to extend its benefits to the fresh cases constantly brought to the notice of the committee.

### CAULS AND COINS.

A scientific explanation of the "caul" given by the trained nurse or certified midwife may satisfy her instructors and also "intelligent mothers," but apparently remains unacceptable to certain fathers in the nineteenth century. District nurses relate that "language" is occasionally bestowed upon them by the male parent, who believes himself a pecuniary loser by the destruction of the membrane to which superstition has attached a monetary value. That the father does not always content himself with verbal abuse of wife and nurse transpired from the recent action of an attendant, who applied for advice to a magistrate because he had been deprived of the chance of preserving the "caul" of his last born child, which he valued at from two to three pounds.

### A WORTHY SOCIETY.

The Ladies' Sanitary Association is a branch of woman's work which should commend itself to all, as cordially as it did to the late Charles Kingsley. He early appreciated the value to the community at large of every woman being conversant with the laws of health, and the names which appear in the annual report of the Association guarantee the soundness of its aims. The subscriptions, however, are in arrears, being last year even below the modest average, and additional members are most earnestly desired. The recently published annual report gives an interesting retrospect of the work done since 1857, and can be obtained of Miss Adams, the secretary, at 22, Berners Street, London. The paragraph on district nursing hardly expresses the opinion of those who agree with Her Majesty the Queen that the poor are entitled equally with the rich to skilled nursing. A perusal of the able articles which appeared in The Hospital of January, 1894, and following weeks, show very plainly the injustice of nursing the sick by means of "comparatively unsuited material having had a paucity of training." This is especially the case in country places where the doctor, whose supervision and instructions are relied upon to balance deficient nursing, is necessarily out of reach daily for many consecutive hours.

### APPRECIATIVE EAST-ENDERS.

On the eve of Miss Vacher's departure from Poplar, a demonstration of a somewhat unique character took place. Representatives of the clubs and trades unions of that thickly-populated locality marched in procession to the Accident Hospital, and, with banners flying, flocked into the fine board-room, which they filled to overflowing. The Hon. Sydney Holland was present, and a beautiful illuminated address, signed by over 150 working men, was handed to Miss Vacher by Sir Donald Currie, who, in a few well-chosen words, expressed the deep gratitude and affection of those for whose welfare, as matron of the Hospital for Accidents, she has done so much. Miss Vacher acknowledged the gift with evident appreciation of this touching testimony to the esteem felt for her by this representative assemblage. The address itself, a beautiful work of art, was accompanied by a packing case to preserve the document during the impending voyage to South Africa. Miss Vacher's future career as head of the nursing department at Kimberley Hospital will be followed with interest and accompanied by heartfelt good wishes from those she leaves behind.

### A CRUSADE AGAINST CAPS.

Criticisms on individual caps are by no means unheard of, but a wholesale condemnation of this form of head-gear is sufficiently uncommon in the present day to make the scene at a recent Camberwell vestry meeting somewhat remarkable. A Miss Brown, it is reported, in accordance with notice, moved that the caps worn by inmates be abolished. Most visitors to workhouses come away with an impression that the white caps and shawls are the pleasantest parts of the women's uniform, care worn old faces are improved by these adjuncts, the hair of an elderly pauper being seldom "a crown of glory." But Miss Brown's objection to caps as "badges of servitude" is deep-rooted, and she also, on the grounds of expense, takes exception to the caps of the nurses; in fact, she does not appear to see a single redeeming feature in those worn to-day. An argument was advanced in favour of doing away with the caps of the inmates on account of the Local Government Board not sanctioning the hire of extra laundry women; but might not the inmates be encouraged in laundry work, which is a necessary and profitable industry? If nurses' caps are abolished on such grounds, aprons, cotton dresses, and underclothing may also be eventually reduced until materials warranted not to wash will be alone in demand. Before condemning it would be well to consider the uses as well as abuses connected with the wearing of caps.

### NATIONAL HEALTH SOCIETY.

In the unavoidable absence of Her Royal Highness Princess Christian at the annual distribution of medals and certificates granted by the National Health Society, the presentation was performed by the Duchess of Westminster. Mr. Arthur Arnold was in the chair, and the meeting, which was largely attended, was held at Grosvenor House on May 11th.

### ROYAL BRITISH NURSES' ASSOCIATION.

Her Royal Highness Princess Christian was present at the adjourned general meeting of the association, which was held on Friday, May 10th, at the society's rooms. The reappearance of the president at 17, Old Cavendish Street, was enthusiastically received by the members present, amongst whom were a large number of medical men specially noticeable. The post of hon. secretary, recently resigned by Mrs. Spencer, has been filled by Mrs. Dacre Craven.

### PRACTICAL MISSIONARIES.

Missionaries early discovered that "some knowledge" of medicine added materially to their usefulness, and now qualified medical men are, happily, associated with many missions. In the same way "some knowledge" of nursing was found to be absolutely essential for women who aspired to mission work abroad, and by degrees the necessity for fully trained nurses has become acknowledged. Yet another sign of practical progress is now observable, classes being held to instruct future missionaries in invalid cooking. The obvious value of such knowledge to women in isolated districts where their patients' comfort lies entirely in their hands was realised by those who inaugurated this addition to the Mildmay scheme of training missionaries.

### DISTRICT NURSING AT HAVERFORDWEST.

At the Temperance Hall at Haverfordwest a meeting was held lately in the interest of the Nurse Fund and it was agreed that every effort should be made to secure the continued services of the nurse who has proved so valuable in the district during the last two years. The work appears to be extremely heavy, for the report read by Archdeacon Hilbers stated that on many occasions last winter the nurse had worked for seventeen or eighteen hours a day. It appears as if an additional nurse would find plenty of occupation, and probably those who have the scheme at heart will not only collect a sufficient sum to support the permanent nurse, but will secure a balance to pay for temporary nursing help when circumstances require it.

### ST. PATRICK'S HOME.

Excellent has been the work of the trained nurses sent out from St. Patrick's Home, Dublin, to tend the sick poor in their own homes. Although in one of the very simple rules which govern the establishment, it is stated that "the institution shall be conducted in accordance with the principles of the Church of Ireland," yet "the services of the nurses are given to all, irrespective of religious creed." The Lady Ardilaun is the patroness, and the Archbishop of Dublin is president of the association, which is in receipt of a special grant from the Queen's Jubilee Institute. The funds need additional support to enable them to keep pace with the steady increase in the work of the nurses. Miss Dunn, general superintendent of the Queen's Jubilee Nurses in Ireland, gives valuable testimony to the work done from St. Patrick's Home, and also speaks of the influence which these trained district nurses are found to exercise in isolated spots where through them the people are "raised, civilised, improved, all round." The nurses work under proper medical supervision, and their services are restricted to the sick poor who cannot pay for the attendance they so sorely need and so warmly appreciate.

### INDIRECT PAYMENT FOR HONORARY (?) SERVICES.

It is often asked, Does this or that person make anything out of his philanthropic work, and, if so, how is it done? This question cannot be answered by any one who has not the most intimate knowledge of the inner working of the particular charity or association with which the person inquired about may be connected. Where this knowledge is wanting it is charitable to hope that any services rendered are given from the purest motives. In these columns we have always advocated the privilege of free whole-hearted personal service in good work. Unfortunately, there is a belief abroad that the services of individuals may be secured for committees and the *honorary* management of certain charities or associations by offering indirect payments which are euphoniously described as "expenses out of pocket." We venture to protest most strongly against the payment of any such expenses in such a form as to become in any way a profit to the individual. If men and women in this country to-day cannot give of themselves in the shape of a little personal service by taking part in the gratuitous management of our charities and associations, then they had better refrain altogether from the work. We would earnestly urge our readers to decline to be connected with any institution, society, or association which directly or indirectly offers payment for any services which may be given in the circumstances indicated.

### WOMEN STUDENTS IN INDIA.

The establishment of a hostel for the Indian female medical students at the Campbell Hospital need no longer be delayed now that the building fund has been so handsomely augmented by Rs.25,000 from the Begum of Murshedabad. This lady has given further encouragement to women students in India by bestowing Rs.50,000 on Kerbela as an endowment for native students.

### SHORT ITEMS.

Mrs. Hilton, Shore House, Shore Road, South Hackney, is appealing for extra subscriptions to enable her to carry on her crêche and homes without reducing the field of work.—The decision to have only two beds at the Barry Cottage Hospital has been recently altered, local liberality having provided two additional ones, which are very acceptable.—The annual report of the Mary Wardell Convalescent Home for Scarlet Fever shows that 145 patients were admitted in the course of last year.—A small convalescent hospital for girls has now been taken by the London Diocesan Council for Preventive, Rescue, and Penitentiary Work. Princess Christian has taken special interest in its establishment, and many gifts have already been received for the house, which will accommodate twelve inmates.—A good report of the West Hartlepool District Nursing Association was placed before the annual meeting.

### Elementary Anatomy and Surgery for Nurses.

By W. McADAM ECCLES, M.B., M.S., F.R.C.S., Lecturer to Nurses, West London Hospital, &c.

### XVII.—THE SYSTEMIC CIRCULATION.

### THE AORTA AND ITS BRANCHES.

IT will be needful to deal in some detail with the systemic circulation, paying especial attention to the chief vessels of the head and neck, and of the extremities. We have already (Lect. XVI.) seen that the commencement of the arterial portion of this system is the large vessel named the aorta which leaves the left ventricle of the heart.

This artery is for descriptive purposes divided into three portions, viz. : (1) The arch of the aorta. (2) The thoracic aorta. (3) The abdominal aorta. The arch and the thoracic part are contained within the cavity of the thorax, while the abdominal portion is within the abdomen. The arch of the aorta is the first portion, and as its name implies it describes a marked curve, passing soon after its origin on the left to the right behind the pulmonary artery, and then altering its course returns across the spine to the left once more. (See Fig. 27.) Some very important vessels, five in number, are given off from this arch. Almost directly after its origin the two coronary arteries already noticed are seen to arise. From the summit of the arch three large vessels spring destined for

FIG. 27.—THE GREAT ARTERIES.

*a*, heart; *b*, arch of aorta; *c*, pulmonary artery; *d*, thoracic aorta; *e*, abdominal aorta; *f*, common iliac artery; *g*, renal artery; *h*, diaphragm; *i*, œsophagus; *j*, trachea; *k*, innominate artery.

the head and neck and the upper extremities. The first, the one most to the right, is called the innominate artery, then comes the left common carotid, and, lastly, the left subclavian. (See Fig. 27.) The distribution of these vessels will be dealt with later on. The rest of the aorta within the thorax runs vertically downwards, somewhat to the left of the spine and behind the œsophagus. On its way it gives off pairs of intercostal arteries which lie in the spaces between the ribs. About the level of the last dorsal vertebra the aorta passes into the abdomen where it is continued as far as the lower border of the fourth lumbar vertebra, at which level, and slightly to the left of the spine, it divides into two branches, the right and left common iliac arteries. Numerous branches to the abdominal walls and to the viscera within the abdomen arise from this portion of the aorta, two very important arteries being the renal, which run one on either side to the kidneys. (See Fig. 27.)

The common iliac arteries are short and soon divide into the external and internal iliacs, the former passing into the lower limbs after a further course along the brim of the pelvis, while the latter enter that cavity to supply the viscera therein as well as to send some branches to the buttocks and perineum.

### THE ARTERIES OF THE HEAD AND NECK.

On either side of the neck runs an artery named the common carotid, which can be easily felt pulsating in most persons. That of the right side springs from the innominate artery, while on the left it is a branch directly from the aorta. At the level of the upper border of the thyroid cartilage—a part of the larynx readily felt in the upper part of the neck—it divides into the external carotid, which supplies the structures of the face, scalp, mouth, and pharynx, and the internal carotid, which is one of the principal arteries to the brain itself. Also passing up the neck, but very deeply placed, being situated in the foramina of the transverse processes of the cervical vertebræ, is the vertebral artery on either side (a branch of the subclavian artery about to be described), which, like the internal carotid, enters the cranium, within which the two vessels join together to form the basilar artery, which, in its turn, bifurcates, and is distributed to the brain after forming a very free anastomosis, or communication with the internal carotid arteries at the base of the brain, known as the circle of Willis. It will thus be seen that the important centre of nervous action is very abundantly supplied with arterial blood.

### Dundee Royal Infirmary.

### THE TRAINING OF PROBATIONERS.

A COURSE of twenty lectures has been given to the probationers at Dundee Royal Infirmary by the Medical Superintendent, Dr. Nathan Raw. In addition to these, special classes have been held for bandaging, application of splints, testing urines and excretions, emergencies, &c. At the conclusion of the course an examination was held as follows:—

### WRITTEN QUESTIONS.

1. Describe as fully as you can the bones forming the pelvis, and those of the lower extremity.
2. Trace the circulation of the blood.
3. Describe the alimentary canal.
4. What is a wound? Describe the various kinds of wounds, and their possible complications. Describe briefly the antiseptic treatment of wounds.
5. A patient is brought to the hospital, having been found by the police lying in the street in an unconscious condition. When admitted he is unconscious. What might be the cause of the unconsciousness? And what treatment would you adopt?
6. State all you can of pneumonia, and make out a temperature, pulse, and respiration chart of a typical case. (*Blank form supplied*).

Maximum marks—twenty for each question.

### Lewes District Nurse Fund.

THE work of the Lewes district nurse extended last year over seven parishes, and 81 cases were attended by her. To these patients 2,537 visits were paid, the services of Nurse Booker being gratefully accepted by them and heartily approved by the doctors under whom she has worked. The board of directors of Lewes Victoria Hospital also appreciate the attention which has been given by the nurse to 36 of their out-patients, and they have expressed their gratitude to the committee of the District Nurse Fund. The appointment of a night nurse to work under Nurse Booker is in contemplation, and will no doubt be of practical value in severe illness in the homes of the very poor. The report of the fund is drawn up after a most commendable fashion, and there seems every prospect of a prosperous future for this young association.

### The Metropolitan Nursing Association.

ANNUAL MEETING.

The nineteenth report of the society hitherto known as the Metropolitan and National Nursing Association, was laid before the annual meeting, which took place at Grosvenor House on the 13th inst., under the presidency of Mr. Caine, M.P. A special general meeting preceded the annual one, for the purpose of passing a resolution to alter the title to "The Metropolitan Nursing Association." Having become the central training home for Queen's nurses, the committee now feel that they must confine its work to the metropolis, leaving the nursing of the provincial and rural sick poor to the Queen's Jubilee Institute. Mr. Caine spoke of the steadily-increasing work of the association, and moved the adoption of the report. This was seconded by Mr. Mocatta, and agreed to. Mr. Rathbone, M.P., remarking on the division of labour which rendered the change of title desirable, explained that in dropping the old name responsibilities were by no means lessened, and the duty of still further raising the standard of district nursing remained with them. He moved a resolution pledging the meeting to support the association. This was seconded by Mr. Timothy Holmes, who said that although he had not much personal acquaintance with the association, he heard on all sides of its good work. He felt that the powers of the institution were inadequate to deal with the enormous field which the metropolis offered, and he considered the association a most deserving one. In supporting the resolution, Mr. D'Arcy Power, referred to hospital wards, in which with every modern appliance, nursing had become elevated to quite a fine art, and he maintained that in the hands of the trained district nurse it took an equally high place. Surgeons could perform operations on patients in poor homes, and carry on treatment in every confidence of success, and Mr. D'Arcy Power bore witness to the appreciation shown by medical men for the skilled assistance of a competent district nurse. The Rev. Dacre Craven moved a formal resolution for the re-election of the council. He also spoke of the encouragement given by the Local Government Board to guardians as regards the employment of nurses. In many cases, he said, the guardians contributed to the Metropolitan or other nursing associations to secure attendance on outdoor parish cases. Mr. Dacre Craven thought there was less enthusiasm now than twenty years ago when this association had been first started, and he therefore pleaded not only for funds but for friends who would give to it personal attention and interest. He regretted the departure of Miss Hughes, for nearly four years superintendent of the Central Home, and testified to her admirable perseverance in making the association better known amongst the doctors as well as to the people themselves. Miss Hughes had been succeeded by Miss Gray, a lady who had had the advantage of working under her for two or three years. Miss Gray received her previous training in the Nightingale School at St. Thomas's Hospital. The meeting concluded with a vote of thanks to the Duke of Westminster for his kindness in lending his house for the meeting. Amongst those present were: The Duchess of Westminster, Mrs. Dacre Craven (who, when Miss Florence Lees, originated the association), Mrs. Cheadle, Mrs. Minet, Miss Peters, Miss Oldham, Miss Petrie, Miss Brierly, and others.

### Wants and Workers.

Could anyone tell me of a home where a lady of thirty given to intemperance could be received on terms not exceeding one guinea weekly?

—*Nurse Mary.*

### Our American Letter.

(Contributed.)

In Philadelphia we learn that a society, of which Miss Emily Wright Bacon is the secretary and treasurer, is being organised under the name of "The American Pension Fund for Nurses." Doubtless the names of the other promoters of this scheme will shortly be published, if it is to be a national and representative movement.

Dr. D. B. Cornell presented the diplomas and badges to the graduates of the Women's Hospital Training School for Nurses at Saginaw, Michigan, early in the year. The ceremony was largely attended by friends of the pupils and others interested in the movement.

On October 1st a three years' course is to be inaugurated at the Brooklyn Homœopathic Training School for Nurses.

Mrs. Bellamy, said to be the first president of the first women's alumnæ association in the world, proved herself an eloquent advocate of the subject which she has so deeply at heart when speaking on it at the Brooklyn Hospital Training School. It was eventually agreed by the nurses present that an alumnæ association should be formed, with Miss Merritt, superintendent of the training school, as first president. The aims of the association include the promotion of social intercourse, the establishment of an annuity fund, to aid members in sickness, &c.

A brilliant reception was given at the New York Hospital last month, by the graduates of the training school for nurses. This training school, which has been in existence nearly sixteen years, ranks high in the United States, and the reputation of the graduates trained under Miss Irene H. Sutliffe is too well known to need comment. The golden badges of the school were distributed to the graduates by Mr. Theodorus B. Woolsey in the course of the evening, the diplomas having been presented at an earlier stage in the proceedings by Mr. Herman H. Camman.

In the May number of the *Trained Nurse* various interesting and instructive articles appear. In the first one, on "Heroic Nurses," it will doubtless be somewhat of a novelty to your English nurses to find that the Orange Training School presents medals for "heroic service" in connection with cases of diphtheria and scarlet fever. It will also no doubt appear somewhat curious to the advocates of trained nurses for outdoor (or district) and private work, that all those to whom the medals were recently presented were pupil nurses, and that they were sent single-handed to cases of malignant scarlet fever. The consistent advocacy of an equalisation of night and day nursing for which The Hospital is remarkable, is apparently a doctrine to which even the most intelligent Americans are not yet reconciled.

In England we hear the skilled nursing of infectious diseases has come to be considered an ordinary duty for which training, remuneration, and adequate assistance are requisite accessories.

### Minor Appointments.

Chorlton Union Infirmary, Manchester.—Miss Edith Hodgson has been appointed Night Superintendent of this infirmary. She was trained at the Monsall Fever Hospital; worked as probationer at the York County Hospital, and as assistant nurse at the Southern Hospital, Manchester; nursing sister at the Cancer Pavilion and Home Hospital, Manchester, and as charge nurse at the City Hospital, Liverpool. Miss Hodgson takes many good wishes with her to her new work.

Leeds Union Infirmary.—Miss Annie Louisa Swiney has been made Night Superintendent of this infirmary. She was trained at the London Hospital, where she afterwards held the post of staff nurse for eighteen months. We wish her every success.

### Letters from Upper Burma.

By Mrs. Ernest Hart.

### II.—LEPERS AND LEPER HOMES.

It is satisfactory to learn that in spite of the amazing superstition and ignorance described in the previous letter as existing in Burma, English medicine and science are steadily making their way. In Rangoon, Burmese girls are successfully trained as nurses in the hospital of the Countess of Dufferin's Fund. The Civil Hospital at Rangoon is a model institution, thanks to the untiring devotion of Dr. Johnstone, and here a large number of patients are treated. In Mandalay two homes for lepers have been established—one by the Wesleyans, under the direction of the well-known missionary, the Rev. Mr. Winston, and the other by the Catholic fathers. I visited both. There are few sights more pitiful than a leper home, for in the present state of science leprosy is not only a loathsome, but an incurable disease. It is very prevalent in Burma, and there are said to be two thousand lepers in Mandalay alone. The disease commences with patches of anæsthesia in different parts of the body; tubercles presently appear in these insensitive spots; tuberosities are developed which later on become inflamed, running sores are formed, and the small bones are ejected from the wounds. The face, the hands, and the feet are the principal parts attacked. The bridge of the nose flattens, the alæ become enlarged, the cheek-bones hypertrophy, the lips swell, and large lumps appear on the surface. In the hands and feet the small bones are gradually exfoliated from suppurating wounds, and the limb is reduced to a stump. Internally there is evidently similar mischief going on, and the patients suffer from dyspepsia, distress, &c. In both the leper homes the arrangements are much the same; the men are separated from the women; each are lodged in large airy bamboo sheds, built on piles, separated from one another, and standing out on the open plain. The patients are under no restraint to stay; but they are not allowed to go about the town, except by express permission. In the Wesleyan Home there were 60 patients; in the Catholic, 140. Dr. Pedley, the medical officer to the Wesleyan Home, considers leprosy incurable; he believes it to be contagious and hereditary. He found that dressing the sores with powdered anti-febrin gave great relief and kept them clean. He cuts down and removes a dead bone when suppuration of the joint commences, and obtains as results rapid healing and cessation of pain. In the Catholic Home, Dr. Kuhne's treatment has been followed. This consists of a bath with rubbing three times a day for half an hour, and a vapour bath once a week. A vegetable diet and strict abstention from fish are also enforced. Where this treatment has been carried out for six months it has given somewhat favourable results. The anæsthesia often disappears, and the patient feels better. The success is thought to be due to the strict cleanliness enforced by the frequent bathing. Count Mattei's drugs have been tried also by the good fathers; but with these, as in Kuhne's treatment, abstinence from fish is insisted upon. Now, the special dainty of the Burmese peasant is Nga-pee, or a paste made of fish dried in the sun, salted, and pressed. Its odour proclaims its condition, but notwithstanding the Burman considers Nga-pee the most savoury and necessary part of his dinner. The Burmese leper cannot be induced to deprive himself of Nga-pee for long, and hence the vegetarian and fish diet treatment has no fair chance in Burma. The fathers consider leprosy to be both hereditary and contagious, and showed me cases of grand-mother, mother, and grandchildren all afflicted with the dire disease. Singing and the services of the Church give the poor lepers some rays of hope and pleasure; they were gathered into the Wesleyan Chapel for me to hear them sing. It was a most pitiful sight to see them squatting on the floor holding Burmese hymn-books in their mutilated hands, beating time with their fingerless stumps, while in harsh discordant voices they shouted Moody and Sankey's hymns with evident satisfaction. Poor things! Their wards were gaily decorated with pictures from the English illustrated papers, due to the thoughtful kindness of Mrs. Pridmore, who often takes her guitar and goes to sing to the lepers. Both homes are supported by small Government and municipal grants, and by voluntary subscriptions. It costs about £5 a year to keep a leper in the home, and £5 could scarcely be better spent by the charitable.

### Care of the Sick in Alexandria and Cairo.

### I.—THE NATIVE HOSPITAL.

Alexandria is a city wherein dwell people of every country in the world, and of every class and condition, who select the part of the town most congenial to their habits and requirements. The Arab quarter is very dirty; the Turkish clean, bright, and comfortable; and the Frank or European division regularly built and elegant, while the villages of the fellahs outside the city are the foulest dens possible.

Naturally epidemics revel here, and hospitals are as much a necessity as in London, so we tried to find out what was being done with the cases of fever, dysentery, ophthalmia, and the constantly occurring accidents in this mixed population of some 200,000.

We found in good working order the Native, the German, the French, the Military, the Jews, and the Greek Hospitals; the three last nursed by English sisters. The Native Hospital is superintended by Dr. Schiess Bey, a Swiss, whose life for the last thirty years has been wholly devoted to this work.

His three great remedies are air, light, and water. The servants and nurses, all natives, are very picturesque in flowing white robes, and they are very deft in their movements.

In addition to the main building, which contains 180 beds, there is at a little distance from it in the garden a pavilion built on arches with a verandah round it. This contains one large ward, with operating room and a small ward of two beds, and very good sanitary arrangements. As far off as possible from these buildings are three tents for infectious diseases, one for leprosy, and another for erysipelas. This hospital takes in every kind of case, and there is a daily average of 200 out-patients, who get advice and medicine gratis.

There are six women's wards, one maternity ward, and a children's division in one part of the main building, the men occupying another side. The midwife is a highly picturesque figure in a cotton dress of many colours, plenty of jewellery, and a long white veil. There were about a dozen black or brown little creatures with dark eyes and woolly heads, these were in charge of a native woman. We asked if the Arabs were patient under suffering, and the doctor said they would undergo operations without a murmur, but they could not endure long continued pain. He found them a most grateful people. That the sick are well cared for is evident, as only 9 per cent. die, 4 per cent. of these deaths being from operations. One nurse usually has the care of ten patients.

On the men's side we noticed the prisoners' ward, with heavily barred doors and windows and a soldier stationed outside. It seemed sad to see them thus guarded, even in extreme illness. The wards everywhere appeared fresh and wholesome; the ophthalmic being the only one where light was not freely admitted.

The food seemed good, and the pharmacy well stocked. The laundry is managed by two Arabs, who do not rub the clothes with their hands but stamp on them with their feet in a huge bath to a sort of rythmical march.

### Everybody's Opinion.

[Correspondence on all subjects is invited, but we cannot in any way be responsible for the opinions expressed by our correspondents. No communications can be entertained if the name and address of the correspondent is not given, or unless one side of the paper only be written on.]

### A WARNING.

"A NURSE" writes: I had an unusual experience a few days ago when I was walking alone, wearing uniform, in a rather crowded part of North London, and I was stopped by a respectably dressed man. After apologising for speaking he asked me if, as a favour, I would go and see his wife, who was taken ill. He then pointed vaguely towards a house in a side street near by. The story struck me as a clever trick, for the man's manner seemed too calm for a genuine case. He was not in the least agitated, nor did he appear distressed when I refused to go with him. Was I right in believing it a fraud? Will you kindly insert this in your columns to put nurses on their guard against similar requests?

### TRAINED NURSES AND ASYLUM ATTENDANTS.

NURSE JESSIE writes: In some late numbers of THE HOSPITAL I have seen the advertisement of the Medico-Psychological Association with respect to examinations in nursing for attendants on the insane. In another column I find advertisements for matrons for asylums who must be trained nurses, with or without asylum experience. What is the use of asylum attendants going in for this examination if trained hospital nurses with no experience in the nursing of the insane are also eligible for the posts of matrons and head attendants? Many attendants work hard for years and give the best years of their lives to attaining proficiency in the care of the insane, and some pass the medico-psychological examination and get certificates. In spite of this they are debarred from holding the better appointments in the asylums and among the patients to whom they have been so long accustomed. It is often said that asylum attendants are rough and not at all what they should be; and surely it is not to be wondered at when there is so very little chance of promotion from the ranks. No doubt many other head attendants could bear me out in saying they have found that the best attendants in any asylum soon go off to be trained as hospital nurses, because they see no chance of getting on in asylum work. Very few of them ever come back to tend the insane. In view of the best management of asylums, it is doubtful if the introduction of trained nurses is beneficial, unless they have also had asylum experience. The care of the insane requires tact and as much knowledge as that needed by any nurse in a general hospital. So many asylum superintendents are now training attendants in a regular way it should not be necessary to draw upon hospitals for matrons and head attendants.

[Our correspondent fails to realise that many excellent attendants, as well as trained nurses, are unsuited to hold positions as heads of departments because they lack the peculiar gifts which such posts demand. No doubt many nurses and attendants have confidence in their own administrative abilities, but this belief hardly in itself constitutes efficiency. It is desirable that other people should also recognise the possession of these gifts. A large experience has proved to us that ability and merit secure sooner or later full recognition, and that those who remain "in the ranks" are chiefly women whose capacity fits them for useful but subordinate positions. Our correspondent's remarks on training show the truth of our own opinion as to the advantage of at least one year's general hospital experience forming part of every asylum attendant's training. The argument that asylum attendants are "rough and not at all what they should be," because there is so very little chance of promotion, is illogical. Certainly such a statement, if accurate, would seriously damage the cause espoused by our correspondent. We are, however, unwilling to agree in this view of asylum attendants, many of whom we know, esteem, and respect. The improvement in the treatment of the insane is most encouraging to those who have their welfare at heart, although something yet remains to be done before diseased minds share equal advantages with diseased bodies. We are inclined to believe even now that an educated head-attendant who had had some general training, being, moreover, a good housekeeper and administrator, would find herself at any rate "one of the selected candidates."—ED. *T.H.*]

### NURSES' EARNINGS.

THE SECRETARY OF THE DEVON AND EXETER HOSPITAL writes: I note in your issue of the 11th your paragraph in reference to the earnings of our nurses. We have tried to get them to join a pension fund, but only a very few will do so. We have, therefore, adopted a "share system," somewhat on the lines of the Guy's and Brighton Hospitals, and the nurses are delighted with this recognition of their work. We put aside a sum not exceeding 25 per cent. of the net profits, and some of the nurses received over £12, and the lowest over £2. I think the plan will work well, but as this is the first year anything has been done in this matter, it is more or less an experiment.

[Is the pension payment compulsory?—ED. *T. H.*]

### ROYAL NATIONAL PENSION FUND.

"NURSE A." writes: I should like to make known through THE HOSPITAL what a great boon the Pension Fund has been to me in a time of illness lately passed through. I joined the Fund for pension, but not for sick pay, and had been unable to put by anything for a time of sickness. I applied to the Benevolent Fund for assistance to enable me to recruit my strength after leaving hospital. The Committee very kindly not only paid all expenses to a home at the seaside, but also suggested my overdue premiums to the Pension Fund should be paid up, so that I might make a fresh start free from pecuniary difficulties. I sincerely hope that this letter may induce many nurses to join the Fund who have hitherto scarcely given a thought to the rainy day that must come sooner or later. If nurses could but realise what a friend the Pension Fund proves to them they would not delay to join it.

### RAYNAUD'S DISEASE.

"NURSE EDITH" writes: Can you give me any particulars of symptoms and treatment of Raynaud's disease. Also of a treatment of rheumatism, in which the diet consists solely of toast, minced beef, and hot water?

[Raynaud's disease is a condition of spasmodic contraction of the arterioles of the extremities, usually commencing in the fingers, whereby the circulation is so impeded as to cause a temporary "deadness" or lividity of the part, or is so obstructed as to cause actual gangrene. The treatment can only be deduced from an individual investigation of each case. It seems quite certain that the spasm is due to a reflex irritation of some kind, and although much may be done by avoidance of cold and by other means to lessen the spasmodic tendency a careful search for the excentric irritant is always necessary. The so-called "beef-steak and hot-water cure" is to a large extent a matter of feeding on animal proteids and encouraging elimination by copious draughts of hot water between the meals. Theoretically the food should consist of lean meat, which, however, always contains a considerable quantity of fat. The permission of dry toast is but a concession to the natural craving for a mixed diet. It is a treatment which should not be undertaken unless under the supervision of a medical practitioner.—ED. *T. H.*]

### Where to Go.

CONCERT AT 41, BELGRAVE SQUARE (by permission of Sir James R. Walker), in aid of St. Saviour's Hospital, Osnaburgh Street, on Monday May 27th, at half-past three p.m. H.R.H. the Duchess of Albany has promised to be present.

ANNUAL GATHERING OF THE MARY ADELAIDE NURSES will be held at the Nineteenth Century Art Society, 9, Conduit Street, on Wednesday, May 22nd, at eight p.m.

SOCIETY FOR PROMOTING EMPLOYMENT OF WOMEN, 22, BERNERS STREET, LONDON.—Meeting at four p.m. on Monday, May 27th.

### With St. Peter's Society to Meiringen.

From close rooms to the pure air of Switzerland; from ceaseless watching by sick beds to the magnificent repose of the eternal hills—no change can be more complete for the tired nurse than one of this sort—to surroundings completely different from those of daily life. It will be a revelation to many, as it was to us, that the cost of travelling in large numbers is so much less than solitary wanderings; a party of fifty or a hundred may, by going together, enjoy a holiday for the comparatively small sum of eight pounds a head. In the hope of making this known to nurses generally I crave a little space in the pages of The Hospital to tell of a happy fortnight spent in peaceful Meiringen. For conductor we had the Rev. J. Horsley, and for our *nom de voyage* "St. Peter's Society of Walworth;" our rule of life, *fais ce que tu voudras*; and our baggage, only such things as we could carry in our hands. This rule about the luggage and the simple proviso that the whole party keep together both going and returning—these are the only conditions imposed by railway and steamboat companies who issue "Society" tickets.

The members of the society met at Holborn Viaduct on June 15th to the number of a hundred, besides numerous friends, come to say "God-speed." Each traveller wore a white rosette on a more or less conspicuous part of the person, according to taste. The tickets for the through journey were distributed at the station, and the party entrained for Dover. The June sun poured down on the smiling Kentish fields as the train whirled by, and the labourer straightened his crooked back for an instant to glance up; and on, on we go till Dover is reached. The Ostend boat, as everyone knows, is no distance from the train. All embarked at once. The feeblest sailor could not complain of that glorious calm sea.

Luncheon on board made us feel at home and emboldened to take a little exercise on deck. This was done with varying success, a look of complete self-satisfaction marking the passengers who traversed the boat from end to end without accident. A little voyage of three hours to Ostend, and then the train was found waiting impatiently. Here an amusing incident occurred, though the humour of it did not appear till afterwards. Being somewhat in the rear of the party, we observed with surprise that those in front, instead of entering the train, paused, and stood in a wavering, undecided fashion on the platform. We soon discovered the reason. On each carriage for the greater length of the train was the legend "Engaged." The modesty of St. Peter's Society prevented its appropriating the seats, and there were no others. So the "society" fumed and raged, lost its temper, and its luggage, and—its head. Our conductor, to whom we looked for counsel and advice, had vanished—chaos reigned. A few bold spirits, regardless of consequences, got into the carriages, and then proceeded to defend the doors against all comers, doubtless in the hope of having their friends with them. Then our conductor appeared and briefly ordered his flock to get in, and the Society of St. Peter put itself into the train as best it could, when it dawned on darkened minds that those formidable labels on the windows referred to the society itself.

Now began a night of misery. The discomforts of that memorable time would have been escaped if all members of the party had kept strictly to hand-baggage instead of invading the carriages with bags, parcels, boxes, wraps, and hold-alls, enough to fill a luggage van! Parties had been broken up in the confusion of the start. Husbands were at one end of the train, wives at the other, their wraps and eatables somewhere in the middle. Ladies were left with whisky and cigars; their menkind in the next carriage perhaps reduced to milk and biscuits. In one carriage a lamp smoked badly, and smelt worse, causing violent language, anguish of mind and real alarm to the nervous.

But the night came to an end, as all nights will, and the woes were speedily forgotten in the superb morning that followed. At Basle there was breakfast and a sorting of ourselves and our belongings, and then the train for Lucerne. From thence by the mountain line (changing at Lucerne) to Bruning. And now we felt we were really in Switzerland. Advantage was taken of our twenty minutes' wait at Bruning to distribute the *bulletins d'arrivée*, or bed room tickets. Thanks to this masterpiece of management on the part of our conductor, all confusion was saved on arrival at Meiringen; a state of things appreciated highly. After leaving Bruning we gently creep up the mountain side, passing within a hundred yards of where the little railway starts on its way up mighty Pilatus. Then down into the valley again to Meiringen where the journey ended in the Hotel du Sauvage, the largest and best in the place—airy, luxurious, and well-appointed in all possible ways.

(*To be continued.*)

### Appointments.

[It is requested that successful candidates will send a copy of their applications and testimonials, with date of election, to The Editor, The Lodge, Porchester Square, W.]

Shipley Infectious Hospital, Stony Ridge.—Miss Rebecca I. Miles has been appointed Matron of this hospital. She was trained at the Bradford Infirmary, Bradford Fever Hospital, and Queen Charlotte's Hospital by the Bradford Nurses' Institution, which she entered as a probationer in 1882. Miss Miles takes with her the cordial good wishes of many friends.

Limerick County Infirmary.—Miss Frances Mayne has obtained the post of Matron in this infirmary. She was trained at Steeven's Hospital, Dublin, for the Nursing Institution, Usher's Quay, and took private cases for two years. She was then appointed charge nurse at King's County Infirmary, Tullamore; afterwards head nurse in Limerick County Infirmary. This position is now combined with that of matron. Miss Mayne has already done much towards re-organising the nursing, and her work has been highly appreciated. We congratulate her on her promotion.

### Notes and Queries.

The contents of the Editor's Letter-box have now reached such unwieldy proportions that it has become necessary to establish a hard and fast rule regarding Answers to Correspondents. In future, all questions requiring replies will continue to be answered in this column without any fee. If an answer is required by letter, a fee of half-a-crown must be enclosed with the note containing the enquiry. We are always pleased to help our numerous correspondents to the fullest extent, and we can trust them to sympathise in the overwhelming amount of writing which makes the new rules a necessity. Every communication must be accompanied by the writer's name and address, otherwise it will receive no attention.

### Queries.

(142) *Holiday.*—Will you kindly tell me if there is a holiday home connected with the Y.W.C.A. in London?—*District Nurse.*

(143) *Recognised Training.*—Please tell me what this means. Does a large workhouse hospital where a three years' course of training is given, count? The matron is properly trained herself.—*N. W. and others.*

### Answers.

(142) *Holiday* (*District Nurse*).—You had better apply to the secretary at the society's headquarters, 316, Regent Street, London. They have cubicles there and would give you information as to their other houses.

(143) *Recognised Training* (*N. W. and others*).—We certainly consider a workhouse infirmary affords recognised training when the matron is herself a fully-trained nurse, and lectures followed by examinations are given to the probationers. The three years' course of training has been introduced into some infirmaries recently, and in others has obtained favour for a long time. We do not consider that training in a "special" hospital only, constitutes complete training, although you will find some associations accept it as such. If you wish to get thoroughly trained, avoid special hospitals of all kinds until you have gone through the regular course of general nursing.

## Figures and Tables

**Fig. 27 f1:**